# Alfaxalone improved in acute stress‐induced tactile hypersensitivity and anxiety‐like behavior in mice

**DOI:** 10.1002/npr2.12233

**Published:** 2022-02-04

**Authors:** Kazumi Yoshizawa, Saki Ukai, Junpei Kuroda, Tsugumi Yamauchi, Daisuke Yamada, Akiyoshi Saitoh, Satoshi Iriyama, Shoichi Nishino, Satoru Miyazaki

**Affiliations:** ^1^ Laboratory of Pharmacology and Therapeutics Department of Pharmacy Faculty of Pharmaceutical Sciences Tokyo University of Science Chiba Japan; ^2^ Laboratory of Pharmacology Department of Pharmacy Faculty of Pharmaceutical Sciences Tokyo University of Science Chiba Japan; ^3^ Laboratory of Quantum Information Dynamics Department of Information Sciences Faculty of Science and Technology Tokyo University of Science Chiba Japan; ^4^ Fujimic, Inc Tokyo Japan; ^5^ Laboratory of Bioinformatics Department of Medical and Life Science Faculty of Pharmaceutical Sciences Tokyo University of Science Chiba Japan

**Keywords:** allopregnanolone, anxiety, hyperalgesia, physical stress

## Abstract

Stress has been shown to affect brain activity and exert potent and complex modulatory effects on pain. Several behavioral tests have shown that acute stress produces hyperalgesia, depending on the stress conditions. In the present study, we investigated the effects of single restraint stress on the tactile threshold and anxiety sensitivity in mice. Mice were evaluated for the tactile threshold using von Frey filaments and for anxiety sensitivity using the elevated plus maze (EPM) test. Tactile thresholds were lowered by both 2 and 4 hour of restraint stress, but anxiety‐like behaviors were observed only after 4 hour of restraint stress in the EPM test. In addition, we found that alfaxalone, which is a positive allosteric modulator of the γ‐aminobutyric acid (GABA)_A_ receptor, prevented restraint stress‐induced hyperalgesia‐like and anxiety‐like behaviors. These results indicate that GABAergic function appears to be critical in the regulation of physical stress‐induced hyperalgesia and anxiety.

## Abstract 2

Alfaxalone (3 mg/kg, i.p.) reversed the effects of restraint stress, as shown by a significant increase in both the tactile threshold and the percentage of time spent in the open arms of the EPM test.

## Graphical abstract

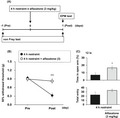

## 1 INTRODUCTION

Stress‐induced exacerbation of pain and the comorbidity of pain with stress‐related psychiatric disorders, such as anxiety and depression, are important clinical issues.^1^ A similar observation has been made in animal studies. Our and other studies have previously reported that a mouse model of neuropathic pain exhibited anxiety‐related behaviors 4 weeks after surgery.^2^, ^3^ Furthermore, hyperalgesia has been shown to be caused by acute stress in rodents.^1^

It has been shown that stress can lead to loss of γ‐aminobutyric acid (GABA)‐ergic neural function. For example, in rodent studies, acute stress has been found to induce a transient and rapid decrease in GABA_A_ receptor function,^4^ which is accompanied by a rapid release of neurosteroids, such as allopregnanolone (ALLO).^5^ ALLO, which acts as a positive allosteric modulator of GABA_A_ receptors, exhibits anxiolytic effects. Although the mice that had been restrained for 2 hour did not exhibit anxiety‐like behavior, mice pretreated with finasteride, an inhibitor of 5‐alpha reductase, which is an ALLO biosynthetic enzyme, exhibited anxiety‐like behavior after 2 hour of restraint. These findings suggest that ALLO biosynthesis contributes to stress resistance.^6^ In contrast, previous report has shown that exposure to 4 hour of restraint stress modified the mouse behavior in the EPM test, decreasing the exploration of open arms.^7^

Stress‐induced hyperalgesia was abolished by the administration of GABA_A_ receptor agonist diazepam, suggesting that the reversal of hyperalgesia was due to the anxiolytic properties of the drugs.^1^ On the other hand, we previously reported that diazepam decreased the grip strength dose‐dependently in the grip test, whereas the ALLO mimetic drug alfaxalone did not affect the muscle tone of the mice.^6^ Thus, alfaxalone is a promising candidate for anxiolytic without benzodiazepine‐like muscle‐relaxant effects.

In the present study, we investigated the effects of single 2 or 4 hour restraint stress on the tactile threshold and anxiety sensitivity in mice. Furthermore, we examined the effects of alfaxalone on hyperalgesia‐like and anxiety‐like behaviors induced by restraint stress.

## 2 METHODS

### 2.1 Animals

Male C57BL/6N mice, 7 weeks old at the time of the experiments, were purchased from Japan SLC (Shizuoka, Japan). Animals were housed in cages in groups of six and given free access to food and water. The room temperature was controlled (23 ± 1°C), and a 12 hour light‐dark cycle (lights on from 8.00 AM to 8.00 PM) was maintained. All experimental protocols were approved by the Institutional Animal Care and Use Committee of the Tokyo University of Science (approval numbers: Y19048 and Y20036). All efforts were made to limit the number and suffering of experimental animals. A minimum of six animals were used for each test.

### 2.2 Restraint stress model

Mice were subjected to a restraint stressor, which consisted of enclosing the animals in a plastic tube (3 cm in diameter and 10 cm in length). A hole in the tip of the tube allowed for breathing, and the mice were restrained for 2 or 4 hour.

The effects of alfaxalone pretreatment on acute restraint stress‐induced hyperalgesia‐like and anxiety‐like behaviors were examined. Pretreatment with alfaxalone (3 mg/kg, i.p.) was performed immediately prior to 2 or 4 hour of restraint stress. The effect of alfaxalone was examined while referring to the pharmacological potency as described previously.^6^

### 2.3 Measurement of tactile threshold

Tactile threshold was measured as previously described.^2^ A series of von Frey filaments (0.07, 0.16, 0.4, 0.6, 1.0, 1.4, and 2.0 g) were applied (Aesthesio^®^, DanMic Global, San Jose, CA, USA). The 50% withdrawal threshold was calculated using the up‐down method^8^ starting with the 0.6 g filament. If a positive response was observed, the next lower force filament was applied, until a change in response was observed. Four subsequent filaments were then assessed according to the up‐down sequence, and the 50% paw withdrawal value was calculated using a previously described method.^9^

### 2.4 Elevated plus maze test

The elevated plus maze (EPM) test was performed as previously described.^6^ Different animals from those for the von Frey test were used in this experiment. The plus maze consisted of four arms: two open arms (6 × 29.5 cm^2^) and two closed arms (6 × 29.5 cm^2^) enclosed by 55 cm high walls. Each arm had a delimited central area of 6 × 6 cm^2^. The entire maze was elevated to a height of 55 cm above the floor and illuminated by a dim light (12 lx) at the end of each open arm. The mice were brought to the corner of the experimental room at least 60 min before the start of the experiment. To begin a test session, the mice were placed in the center of the maze facing one of the open arms. Entry into an arm was defined as the animal placing two front paws over the line marking that area. The number of open‐arm entries, closed‐arm entries, and the time spent in the open arms was recorded during the 5 min test period. The percentage of time spent in the open arm of the maze ([open‐arm time/total test time] ×100), and the total entries (open‐arm entries +closed‐arm entries) were calculated for each animal.

### 2.5 Drugs

Alfaxalone was purchased from MP Biomedicals, Inc (Tokyo, Japan) and was diluted in saline (0.9% sodium chloride) to a volume of 10 ml/kg.

### 2.6 Data analysis

Data were expressed as mean ± standard error of the mean (SEM) and were evaluated by one‐ or two‐way analysis of variance (ANOVA) followed by Bonferroni's multiple comparisons test. Statistical analysis for two‐group comparisons was performed using unpaired t tests with Welch's correction. All statistical analyses were performed using Prism 8 for macOS (GraphPad Software, Inc).

## 3 RESULTS

### 3.1 Restraint stress induces hyperalgesia‐like and anxiety‐like behaviors

**FIGURE 1:**
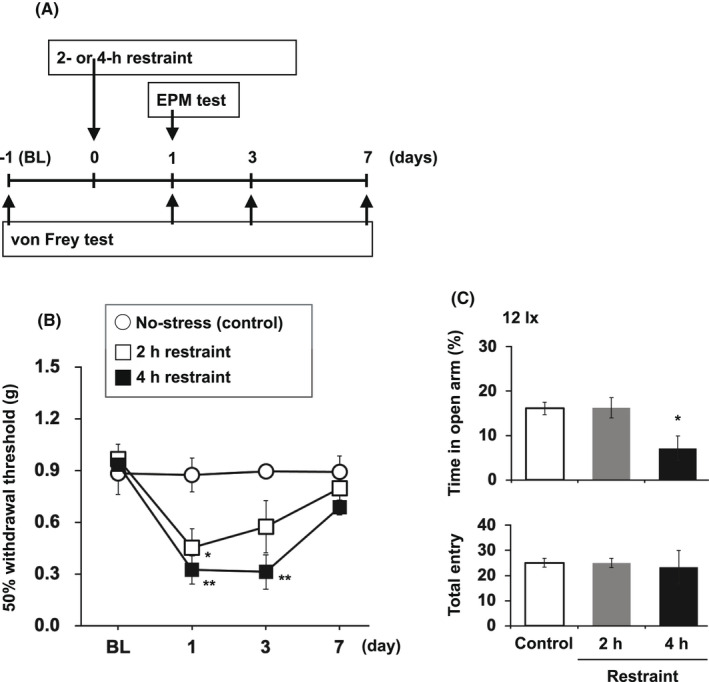
Showing the experimental timeline A. Mice were evaluated for the tactile threshold using von Frey filaments B, and for anxiety sensitivity using the EPM test C, after 2 or 4 h of restraint stress. Each point or column represents the mean ± SEM of six mice per group. *P < .05 and **P < .01 versus the control group. Raw data of Figure 1B and 1C are shown in Tables S1‐S2

Figure 1A shows the experimental timeline. Both 2 and 4 hour of restraint stress caused a transient decrease in tactile threshold in mice (Figure 1B; two‐way ANOVA: column factor, *P* = .0265; time factor, *P* < .0001; interaction, *P* = .0006; Bonferroni's test, *P* < .01 vs. control group). However, while 4 h of restraint stress significantly reduced the percentage of time in the open arm of the EPM test (Figure 1C; one‐way ANOVA, F[2, 15] = 5.508, *P* = .0161; Bonferroni's test, *P* < .05 vs. control group), mice exposed to 2 h of restraint stress did not spend a different percentage of time in the open arms of the EPM test, compared with mice that had not been restrained (Figure 1C).

### 3.2 Preventive effects of alfaxalone by restraint stress

**FIGURE 2:**
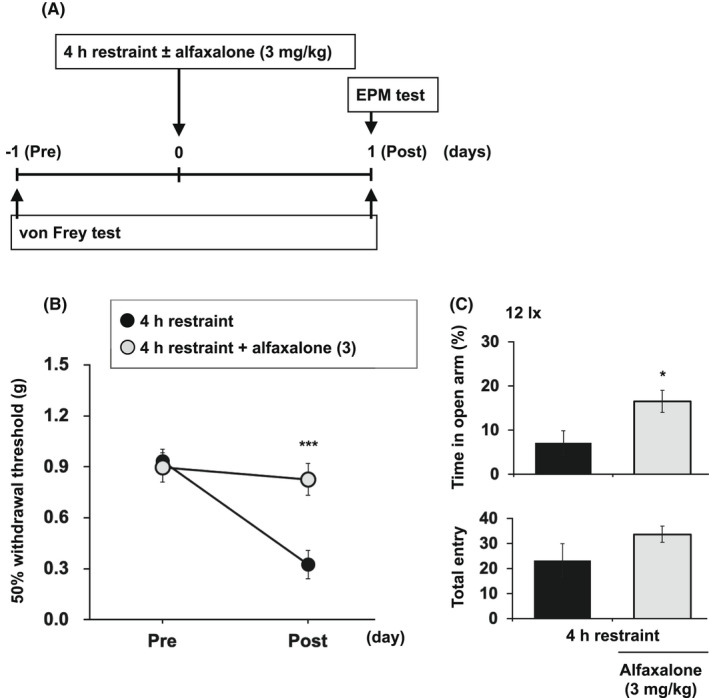
Showing the experimental timeline A. Effects of alfaxalone on the tactile threshold evaluated using von Frey filaments B, and on anxiety sensitivity evaluated using the EPM test C, in stressed mice. Each point or column represents the mean ± SEM of six mice per group. *P < .05 and ***P < .005 versus the restraint alone group. Raw data of Figure 2B and 2C are shown in Tables S1‐S2

Figure 2A shows the experimental timeline. Alfaxalone reversed the effects of restraint stress, as shown by a significant increase in both the tactile threshold (Figure 2B; two‐way ANOVA: column factor, *P* = .0142; time factor, *P* = .0031; interaction, *P* = .0119; Bonferroni's test, *P* < .005 vs. restraint alone group) and the percentage of time spent in the open arms of the EPM test (Figure 2C; *t* test, *P* = .0305).

## 4 DISCUSSION

Stress may exert a bidirectional modulatory effect on pain, eliciting either stress‐induced analgesia or stress‐induced hyperalgesia, depending on its duration and intensity.^10^ Atwal et al^11^ demonstrated that paw withdrawal latency was significantly greater immediately post‐stress in animals that underwent 30 or 60 min restraint than at the pre‐stress time points. Additionally, this stress‐induced analgesia was transient and lasted only 30 minutes. In contrast, in the present study, mice were restrained for 2 or 4 hour periods, and stress‐induced hyperalgesia was observed 24 hour later. Thus, stress‐induced analgesia may have been observed in the mice immediately post‐restraint stress. However, we did not measure whether tactile thresholds were increased immediately after 2 or 4 hour of restraint. This is a limitation of this study.

In the next experiment, we examined the involvement of GABA in hyperalgesia‐like and anxiety‐like behaviors induced by restraint stress. We found that alfaxalone prevented restraint stress‐induced hyperalgesia‐like and anxiety‐like behaviors. Forced swimming stress has been shown to cause hyperalgesia and decrease spinal GABA release, both of which were prevented by pre‐stress treatment with the GABA_A_ receptor agonist diazepam.^12^ This effect was blocked by flumazenil, suggesting the involvement of GABA_A_ receptors. Therefore, GABAergic function appears to be critical in the regulation of stress‐induced hyperalgesia and anxiety. Moreover, it has been reported that the analgesic effect of *N*‐palmitoylethanolamine (PEA), the endogenous amide of palmitic acid and ethanolamine, is mediated by ALLO biosynthesis.^13^ Notably, administration of carrageenan and formalin decreased ALLO levels in the pain process, whereas PEA administration increased ALLO content.^13^ These data suggest that the decrease in ALLO biosynthesis may play a role in inflammatory pain, and moreover, identifies ALLO biosynthesis facilitators, such as PEA, as potential treatment for inflammatory pain.^13^ ALLO biosynthesis facilitators have antinociceptive effects not only on inflammatory pain but also on neuropathic pain. Etifoxine, which is known to stimulate ALLO biosynthesis in the spinal cord,^14^ treatment for five consecutive days (50 mg/kg) relieved mechanical allodynia.^15^ In addition to its effect on evoked mechanical allodynia, etifoxine was also shown to exhibit anxiolytic activity under neuropathic pain‐induced anxiety.^15^

Alfaxalone has potent anesthetic activity via GABA_A_ receptors. A previous report showed that administered alone, alfaxalone had no antinociceptive effects at non‐sedative doses and it had no effect on opioids, such as morphine, fentanyl, or oxycodone, antinociception.^16^ Therefore, prevention of hyperalgesia‐like behavior by alfaxalone might be an indirect effect associated with anti‐stress effects rather than a direct antinociceptive effect.

The glutamatergic system contributes to the stress‐induced hyperalgesia. For example, restraint stresses induce glutamate release in the brain regions, such as hippocampus.^1^ Furthermore, water avoidance stress‐induced hyperalgesia was associated with decreased spinal expression of the glial glutamate transporter, and this stress‐induced visceral hyperalgesia was blocked by pharmacological inhibition of spinal N‐methyl‐D‐aspartate receptors.^1^ Previous studies have shown that ALLO^17^ and alfaxalone^18^ decrease glutamate release in discrete brain regions including the hippocampus and cerebrocortex. On the other hand, Spigelman et al^19^ have found that behavioral and physiological sensitivity to alfaxalone is attenuated in δ subunit knockout mice. In the spinal cord, the α2, α3, α5, β3, γ1, and γ2 mRNAs are present at high levels, but transcripts for α1 and δ are not detected.^20^ Therefore, the prevention of hyperalgesia‐like behavior by alfaxalone may contribute to the inhibition of glutamate release in the brain rather than the spinal cord.

In conclusion, we clearly demonstrated that GABAergic function may contribute to restraint stress‐induced hyperalgesia‐like and anxiety‐like behaviors in mice.

## CONFLICT OF INTEREST

This work was supported in part by a grant from Fujimic, Inc (Tokyo, Japan). Mr Shoichi Nishino is the employee of Fujimic, Inc

## AUTHOR CONTRIBUTIONS

KY designed the experiments and wrote the manuscript. SU and JK conducted some of the in vivo studies. TY and DY provided scientific and technical advice. AS, SN, SI, and SM have supervised the overall projects contributed to design of the protocol and story of the manuscript. All of the authors discussed the results and commented on the manuscript. All authors have critically reviewed content and approved final version submitted for the publication.

## APPROVAL OF THE RESEARCH PROTOCOL BY AN INSTITUTIONAL REVIEWER BOARD

N/A

## INFORMED CONSENT

N/A

## REGISTRY AND THE REGISTRATION

N/A

## ANIMAL STUDIES

All animal experimental protocols were approved by the Institutional Animal Care and Use Committee of the Tokyo University of Science (approval numbers: Y19048 and Y20036).

## Supporting information

Table S1‐S2

## Data Availability

The data that supports the findings of this study are available in the supplementary material of this article.
